# Transmission of Induced Chromosomal Aberrations through Successive Mitotic Divisions in Human Lymphocytes after *In Vitro* and ﻿*In﻿ Vivo* Radiation

**DOI:** 10.1038/s41598-017-03198-7

**Published:** 2017-06-12

**Authors:** Akram Kaddour, Bruno Colicchio, Diane Buron, Elie El Maalouf, Eric Laplagne, Claire Borie, Michelle Ricoul, Aude Lenain, William M. Hempel, Luc Morat, Mustafa Al Jawhari, Corina Cuceu, Leonhard Heidingsfelder, Eric Jeandidier, Georges Deschênes, Alain Dieterlen, Michèle El May, Theodore Girinsky, Annelise Bennaceur-Griscelli, Patrice Carde, Laure Sabatier, Radhia M’kacher

**Affiliations:** 1 Laboratory of Radiobiology and Oncology and PROCyTOX, DRF, CEA, Paris-Saclay, France; 2 Tunis El Manar University, School of Medicine, Tunis, Tunisia; 3 Laboratoire MIPS Groupe IMTI Université de Haute-Alsace, Mulhouse, France; 4 Pole Concept, Paris, France; 5 APHP-Hopital Paul Brousse Université Paris Sud/ESteam Paris Inserm UMR 935, Villejuif, France; 6 MetaSystems GmbH, Robert-Bosch-Str. 6 D-68804, Altlussheim, Germany; 7 Service de Génétique Groupe Hospitalier de la Région de Mulhouse et Sud Alsace, 68070 Mulhouse, France; 8 Nephrology Department, APHP-Hopital Robert Debré, Paris, France; 9 Department of Radiation Oncology, Gustave Roussy Cancer Campus, Villejuif, France; 10 Department of Hematology, Gustave Roussy cancer Campus, Villejuif, France; 11 Cell Environment, Paris, France

## Abstract

The mechanisms behind the transmission of chromosomal aberrations (CA) remain unclear, despite a large body of work and major technological advances in chromosome identification. We reevaluated the transmission of CA to second- and third-division cells by telomere and centromere (TC) staining followed by M-FISH. We scored CA in lymphocytes of healthy donors after *in vitro* irradiation and those of cancer patients treated by radiation therapy more than 12 years before. Our data demonstrate, for the first time, that dicentric chromosomes (DCs) decreased by approximately 50% per division. DCs with two centromeres in close proximity were more efficiently transmitted, representing 70% of persistent DCs in ≥M3 cells. Only 1/3 of acentric chromosomes (ACs), ACs with four telomeres, and interstitial ACs, were paired in M2 cells and associated with specific DCs configurations. In lymphocytes of cancer patients, 82% of detected DCs were characterized by these specific configurations. Our findings demonstrate the high stability of DCs with two centromeres in close proximity during cell division. The frequency of telomere deletion increased during cell cycle progression playing an important role in chromosomal instability. These findings could be exploited in the follow-up of exposed populations.

## Introduction

The scoring of induced chromosomal aberrations (CAs) in blood T-lymphocytes is an important tool for the estimation of the absorbed dose in biological dosimetry^1^ and a reliable biomarker that can predict cancer risk in healthy subjects^2, 3^. The turnover rate of lymphocytes and the transmissibility of CAs are major challenges for the realization of retrospective biological dosimetry after accidental or professional exposure^4–7^ and in the assessment of the associated risk in exposed populations^6, 8–11^. The turnover of lymphocytes is relatively slow, approximately three years^12^, but the transmission of dicentric chromosomes (DCs), specific markers of irradiation, is theoretically reduced by about a factor of two per cell cycle, making them an unstable CA. According to the theory of Carrano and Heddle^13^, a DC has an equal chance of pulling free or producing a lethal anaphase bridge during anaphase. Several studies^14–19^ have documented the transmission of DCs, supporting this theory, using conventional techniques (FPG) or fluorescence *in situ* hybridization with centromeric DNA probes and chromosome painting^14, 20, 21^. The mechanism that controls the transmission of centric rings and particularly acentric chromosomes (ACs), remains unclear^15, 22^.

We recently demonstrated that the introduction of telomere and centromere (TC) staining, using PNA probes, renders not only DC and centric ring (CR) scoring more efficient and robust, but also permits the detection of different types of ACs: ACs with four telomeres, resulting from a fusion event generally accompanying the formation of a DC or CR; ACs with two telomeres, representing terminal deletions; ACs without telomeres, representing interstitial deletions, as well as telomere deletions of the chromosome^23–25^.

Here, we demonstrate that TC staining followed by the M-FISH and extended lymphocyte culture time for the analysis of the transmission of unstable CA through multiple mitotic cell divisions measures the transmission of DC and CR with greater efficiency than conventional techniques. We also demonstrate, for the first time, the transmission of different types of ACs and telomere deletions. Using this approach, it was possible to evaluate the persistence of several DC configurations, highlighting the importance of DCs with two centromeres in close proximity. In addition, we validated our findings in a large cohort of patients treated more than 12 years prior by radiation therapy. These improvements permit a more precise estimation of the genotoxic risk immediately after exposure, as well as a long time after. This approach marks a potential new step in the assessment of cancer risk after exposure to ionizing radiation. Telomere deletions are a major aberration found in circulating lymphocytes that passed through three or more divisions. This aberration could be detected only using TC staining.

## Results

### Transmission of unstable CA after *in vitro* irradiation

**Figure 1:**
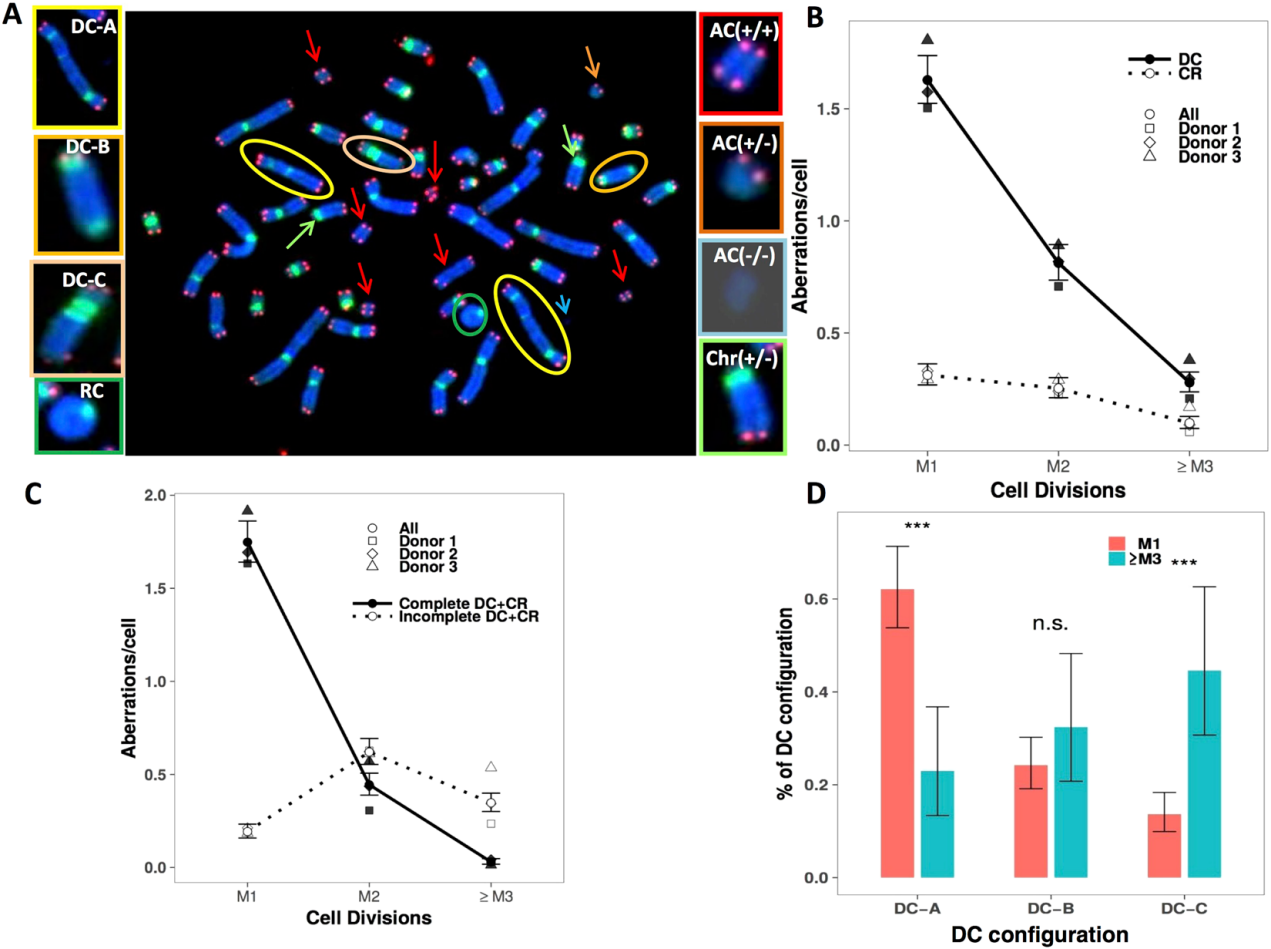
Transmission of DC and CR after γ-ray 4 Gy ^137^Cs irradiation following TC staining. We plotted the mean frequency of CAs for three donors, as well as the individual values for each donor. (**A**) TC staining improved the detection of different DCs configurations when the centromere was in close proximity to or in contact with the telomeres (DC-B) and when both centromeres in close proximity (DC-C). It was possible to easily distinguish centric and acentric rings. We detected AC (+/+) with 4 telomeres resulting from a fusion event generally accompanying the formation of a DC or CR; AC (+/−) with 2 telomeres representing terminal deletions; AC(−/−)without telomeres representing interstitial deletions and telomere chromosome deletion (Chr (+/−)). (**B**) The transmission of DC and CR: The decrease in the transmission frequency of DCs by cell division was greater than that of CRs. (**C**) The frequency of complete and incomplete DC and RC transmission by cell division. (**D**) The frequency of various DC configurations in M1 and ≥M3 cells.

The frequency of induced unstable CAs (Fig. 1A) in circulating lymphocytes of the three healthy donors after 4 Gy γ-ray exposure per cell division and by sampling time are shown in Table 1. The frequency of unstable CAs for each donor is shown in Supplementary Table S1. The proliferation indices for controls and irradiated cells are shown in Supplementary Table S2. The data indicate that 50% of the metaphases of the controls were in M2 after 50 h of culture and most cells had passed through two divisions by 72 h and three or more division (≥M3) by 96 h. We observed a mitotic lag of the exposed lymphocytes, due to irradiation, mostly during M1, and the proliferation indices were comparable between control and irradiated lymphocytes by 96 h of culture.

**Table1:** Frequency of unstable chromosomal aberration through cell division and following 4 Gy γ-ray exposure of circulating lymphocytes using TC staining according cell cycle and time culture.

Cell Division	M1						M2						M3			
Time of culture (h)	50		72		96		50		72		96		72		96	
Scored cells	454		75		31		174		231		101		177		393	
Total DC (/cell)	705	(1,55)	169	(2,25)	38	(1,23)	156	(0,90)	198	(0,86)	57	(0,56)	74	(0,42)	85	(0,22)
Total RC (/cell)	134	(0,30)	28	(0,37)	13	(0,42)	48	(0,28)	57	(0,25)	23	(0,23)	22	(0,12)	34	(0,09)
**Total DC** + **RC** (**/cell**)	**839**	(**1**,**85**)	**197**	(**2**,**63**)	**51**	(**1**,**65**)	**204**	(**1**,**17**)	**255**	(**1**,**10**)	**80**	(**0**,**79**)	**96**	(**0**,**54**)	**119**	(**0**,**30**)
**Total AC** (**/cell**)	**1213**	(**2**,**67**)	**250**	(**3**,**33**)	**66**	(**2**,**13**)	**348**	(**2**,**00**)	**472**	(**2**,**04**)	**148**	(**1**,**47**)	**56**	(**0**,**32**)	**90**	(**0**,**23**)
AC(+/+) (%AC)	840	(69%)	160	(64%)	42	(64%)	217	(62%)	279	(59%)	82	(55%)	23	(41%)	57	(63%)
AC (−/−) (%AC)	261	(22%)	42	(17%)	18	(27%)	103	(30%)	166	(35%)	51	(34%)	28	(50%)	21	(23%)
AC(+/−) (%AC)	112	(09%)	48	(19%)	6	(09%)	28	(08%)	27	(06%)	15	(10%)	5	(09%)	12	(13%)
**Chr**(**+/−**) (**/cell**)	**95**	(**0**,**21**)	**38**	(**0**,**51**)	**18**	(**0**,**58**)	**46**	(**0**,**26**)	**102**	(**0**,**44**)	**35**	(**0**,**35**)	**32**	(**0**,**18**)	**74**	(**0**,**19**)
Complete DC + CR (/cell)	780	(1,72)	162	(2,16)	37	(1,19)	91	(0,52)	104	(0,45)	30	(0,30)	6	(0,03)	11	(0,03)
Incomplete DC + CR (/cell)	59	(0,13)	35	(0,47)	14	(0,45)	113	(0,65)	151	(0,65)	50	(0,50)	90	(0,51)	108	(0,27)

The difference in proliferation indices and cell cycle delays in the three donors may lead to an underestimation the frequency of induced CAs if the metaphases were analyzed at one-time point. We thus evaluated the transmission of CAs through successive metaphases analyzed after 50, 72, and 96 h.

#### Transmission of dicentric and centric ring chromosomes after *in vitro* irradiation

No DCs were observed before irradiation in a total of 1826 scored cells. The transmission of DCs and CRs after 4 Gy of γ-ray exposure, by cell division, is shown in Fig. 1B. The yield of DCs decreased by a 50.12% in passing from M1 to M2 and 65.66% from M2 to ≥M3. The yield of CRs decreased by 19.05% in passing from M1 to M2 and 61.16% from M2 to ≥M3. Moreover, the F-ratio (the ratio of DCs to CRs) was 5.41, 3.35, and 2.68 at M1, M2, and ≥M3, respectively.

The frequency of incomplete DCs and CRs represented 9.93% in M1 cells, 58.3% in M2 cells, and 92.1% in ≥M3 cells. Sequential analysis using TC staining following M-FISH revealed that only 41.7% and 7.9% of DCs and CRs at M2 and M3, respectively, were accompanied by a pair of AC (+/+) (Fig. 1C).

We focused on the transmission of specific DC configurations: DC-A: DCs with two centromeres far apart; DC-B: DCs with the centromere region close to the telomeres, and DC-C: DCs with two centromeres in close proximity. Figure 1D shows the rate of transmission of the various configurations of DCs. The proportion of DC-A of the total ≥M3 DCs was significantly less than that of M1 DCs (p < 10^−5^), whereas the proportion of DC-B of the total M1 and ≥M3 DCs was similar. Nevertheless, there was a significant difference in the proportion of DC-C of the total M1 DCs and that of ≥M3 DCs (p < 10^−6^). In addition, small CRs represented 85% of the total number of CRs observed at M3.

The frequencies of DCs in M1 cells were distributed according to the Poisson distribution (Supplementary Table S3). The distributions of DCs in M2 and ≥M3 cells showed a noticeable loss of randomness upon cell division (Supplementary Table S3).

#### Transmission of acentric chromosomes

**Figure 2:**
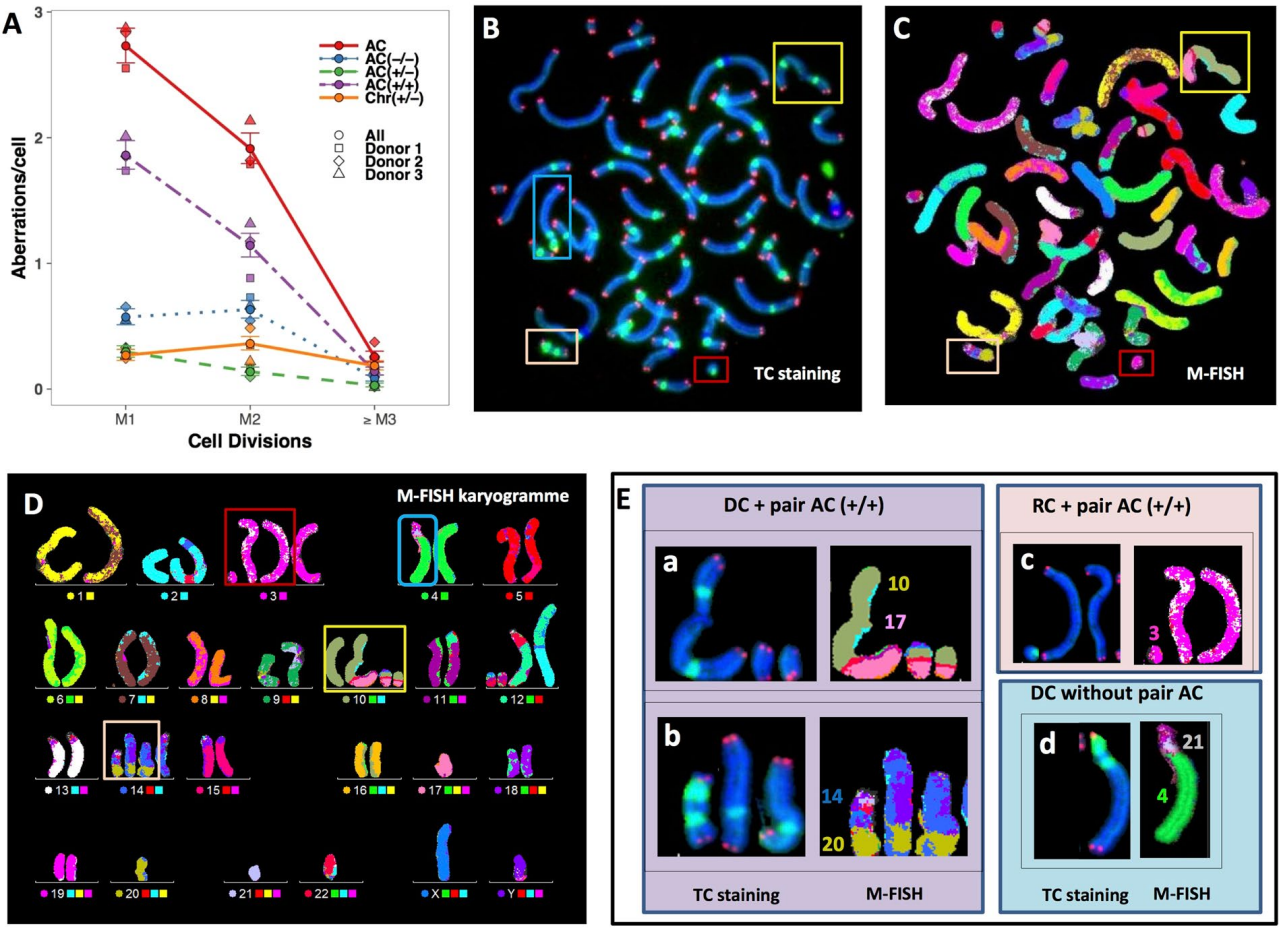
Transmission of different types of ACs. (**A**) The frequency of AC (+/+) and terminal deletions (AC (+/−)) decreased by cell division. The frequency of interstitial deletions (AC (−/−)) and the telomere deletions (Chr (+/−)) increased between M1 and M2. (**B**–**D**) Sequential analysis by TC staining and M-FISH revealed the presence of pairs of AC (+/+) and AC (−/−) associated with DCs and RC (**E**-a) with breakpoints near the centromeric region and the formation of DC(10;17) accompanied by a pair of AC(10;17) (+/+) (**E**-b) The presence of a pair of AC(14;20)(+/+) in M2 cells with centromere sequences, demonstrating that the breakpoints were in the centromeric region and associated with the presence of DC(14;20) with the two centromeres in close proximity. (**E**-c) the presence of small rings characterized by the two breakpoints near the centromeric region associated to the formation of pair AC(3;3) (+/+). (E-d) DC(4;21) without the pair of AC(+/+) was characterized by the breakpoints not localized within the centrometric region.

We observed few ACs in control cells, essentially AC (+/−) and telomere deletions (Chr +/−) in M1 cells. The yields of ACs per cell following 4 Gy irradiation in M1, M2, and ≥M3 were 2.73, 1.91, and 0.25, respectively. The decrease of the total AC frequency was 30% between M1 and M2; the proportion of total ACs in M1 cells was 69.24% AC (+/+), 9.23% incomplete AC (+/−), 21.51% interstitial deletion AC (−/−). The progression of these aberrations as a function of cell division is shown in Fig. 2A. The yield of AC (+/+) and AC (+/−) decreased by a factor of 36.3% and 52.32%, respectively, in passing from M1 to M2. Only the yield of interstitial deletions (AC (−/−)) and telomere deletions (Chr (+/−)) increased with cell division (10.33% and 34.12%, respectively) in all donors. Sequential TC staining (Fig. 2B) and M-FISH (Fig. 2C,D) demonstrated that, only AC (+/+) and AC (−/−) observed in M1 were paired in M2 cells. The breakpoint appears to be near the centromere region (Fig. 2E(a-c) were associated, in most cases, with the formation of a pair of AC (+/+). We also detected the presence of centromere sequences in some pairs of AC (+/+) (Fig. 2Eb) showing that the breakpoint was in the centromeric region. Of note, DC with breakpoint far from centromeric region was not associated to the formation of a pair of AC (+/+) (Fig. 2Ed).

In contrast, sequential analysis demonstrated no pairs of AC (+/−), suggesting the absence of the transmission of this type of AC. The AC (+/−) found in M2 and M3 cells were formed *de novo* and were not transmitted.

### Induced chromosomal aberrations in M1 cells at different times after exposure

**Figure 3:**
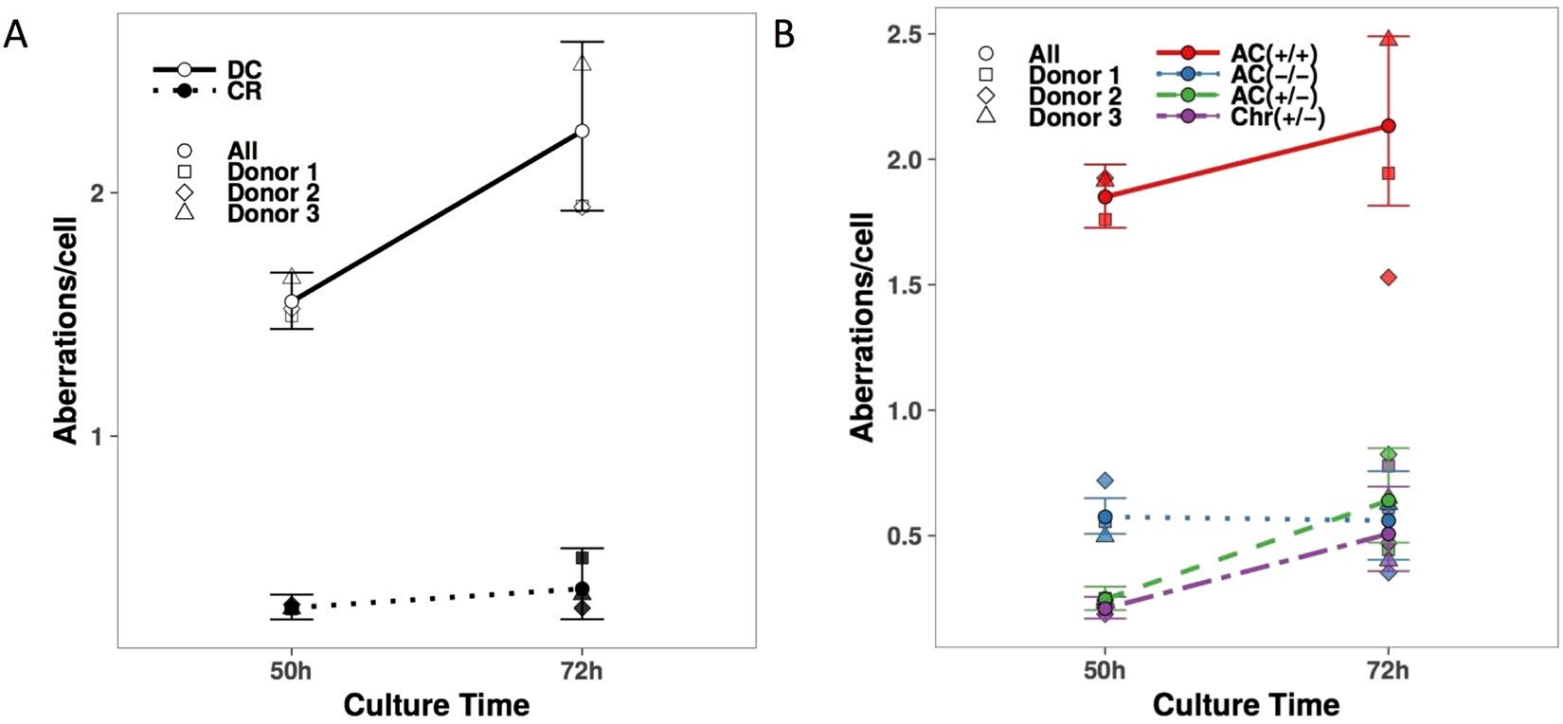
The frequency of CAs of M1 cells after 50 or 72 h in culture after 4 Gy irradiation. (**A**) The frequency of DCs and CRs in M1 after 72 h of culture was greater than that after 50 h of culture. (**B**) The frequency of AC (+/−), AC (+/+), and Chr (+/−) in M1 scored after 72 h of culture was greater than that after 50 h of culture.

Biological dosimetry is based on the scoring of CAs in the first mitosis after 46–50 h of culture after mitogen stimulation. We scored the frequency of CAs in M1 after various times of culture. We assessed the frequency of CAs at 50 and 72 h of culture because all of the cells had undergone more than one mitosis after 96 h. Figure 3A shows a The frequency of DCs was significantly higher (p-value < 10^−5^) for M1 after 72 h than after 50 h of culture, by a factor of 1.45. The frequency of AC(+/−), as well as Chr (+/−), increased with time in culture (Fig. 3B) by a factor of 2.59 and 2.42, respectively (p-value < 10^−7^ and p < 10^−5^, respectively). We did not observe this increase of CAs with time in culture in M2 and M3 cells (Supplementary Figure S1).

### Presence of Giant cells with multiple chromosome aberrations, chromosome pulverization, and telomere aberrations, leading chromosomal instability after *in vitro* radiation

**Figure 4:**
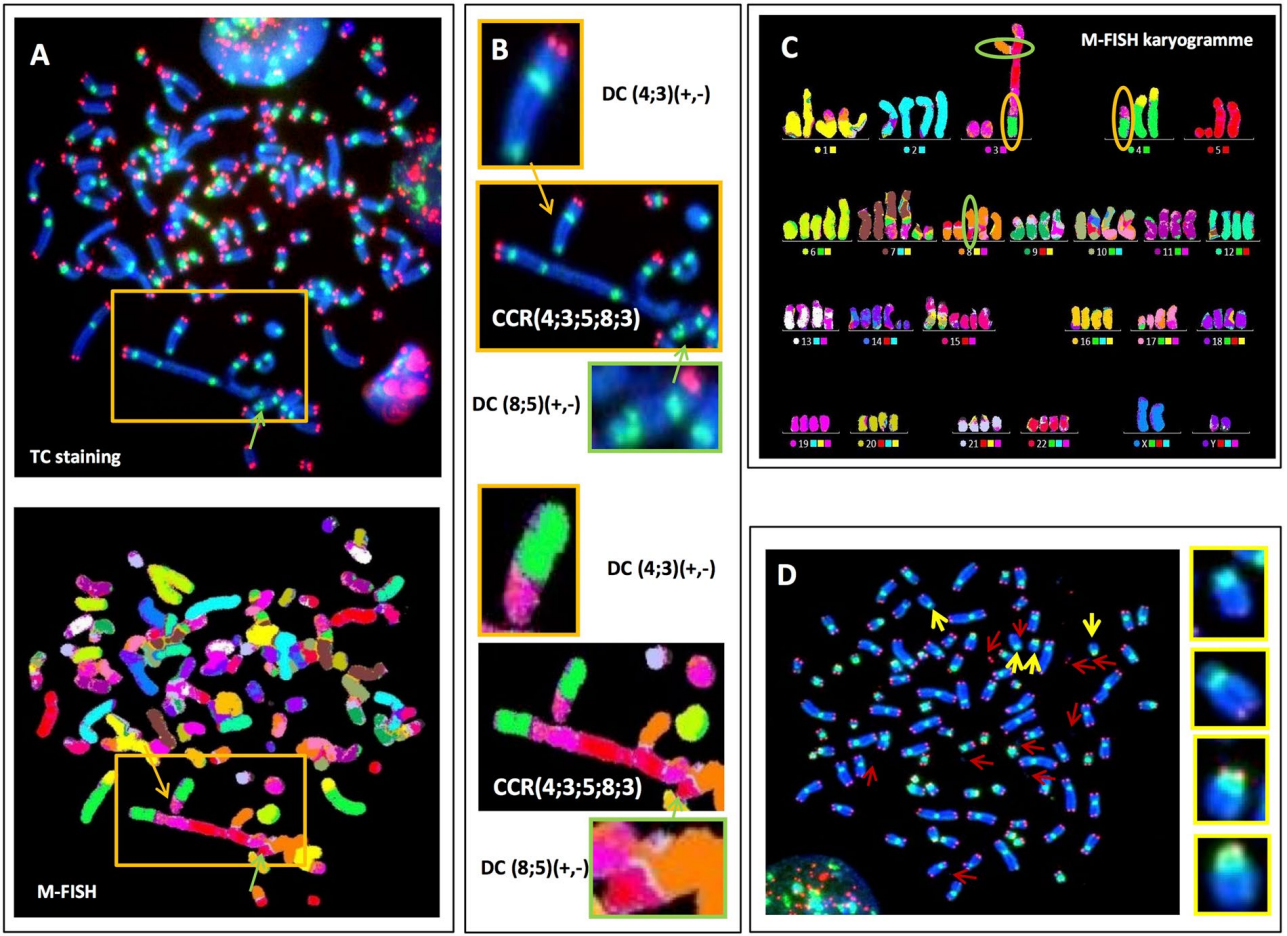
Donor-dependent frequency of giant cells in M2 after TC staining. (**A**) The high frequency of induced CAs, DCs, CR, and ACs (AC (+/+) and AC (−/−)) revealed by sequential analysis using TC staining and M-FISH demonstrates the presence of a pair of DCs, CR, and ACs, indicating replication of these aberrations during cell division. (**B**) Telomere deletion appears to play a major role in the formation of complex chromosomal rearrangements and the progression of chromosomal instability (**C**) the classification of chromosome demonstrated the complexity of induced aberrations (**D**) Giant M2 cell characterized by fewer unstable CAs, but a higher frequency of telomere deletions (yellow arrow). Chromosome pulverization was observed (red arrow).

After 4 Gy exposure, the donor dependent frequency of giant cells was 13% for metaphases scored in M2 and ≥M3 cells (10–17%). Two types of giant cells were observed. The first were characterized by a large number of pairs of DCs, CRs, AC (+/+), and AC (−/−) (Fig. 4A,B. We also observed a high frequency of Chr (+/−) and DC (+/−) with a breakpoint in the pericentromeric region which appears to play a major role in the formation of complex CAs and the induction of chromosomal instability (Fig. 4B,C). The second type of giant cells were characterized by the presence of a high frequency of telomere deletions and chromosome pulverization (Fig. 4D). Telomere dysfunction and telomere deletion were thus a common feature in both types of giant cells.

### Validation of findings in patients treated with radiation therapy

**Figure 5:**
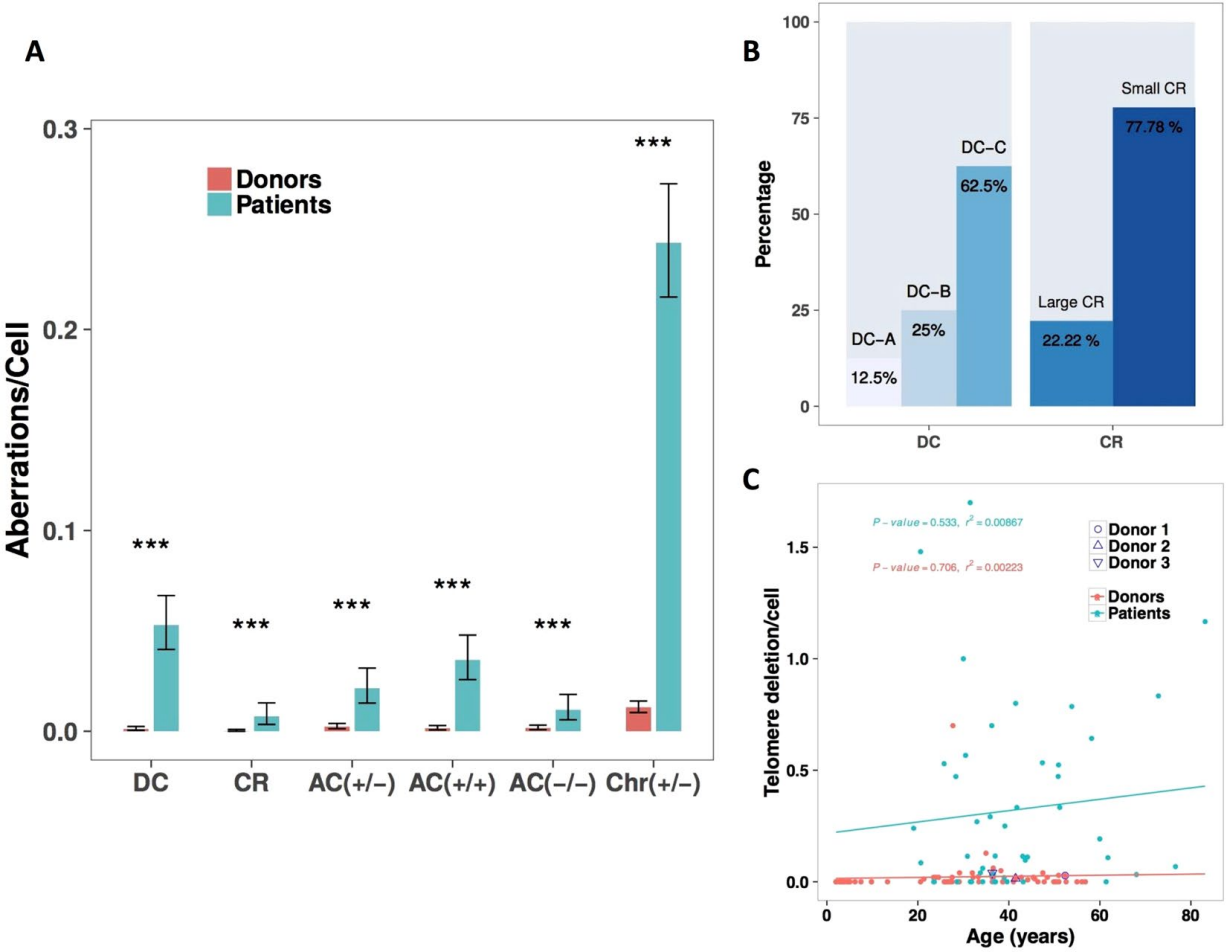
The frequency of CA in circulating lymphocytes of treated patients compared to that of healthy donors. (**A**) significant difference was observed between the frequency of DC (p < 10^−16^), CR (p < 10^−6^), AC(+/+)(p < 10^−16^), AC(+/−)(p < 10^−11^), AC(−/−)(p < 10^−5^) and chr(+/−) (p < 10^−16^), between treated patients and healthy donors (**B**) The prevalence of specific configurations of DC and RC in circulating lymphocytes of treated patients. (**C**) Correlation between telomere deletion of circulating lymphocytes and age of healthy donors and treated patients. The frequency of telomere deletion was not age-dependent in the healthy donors, nor in treated patients. Telomere deletion in lymphocytes of healthy donors is represented by the grey circles and the treated patients by black circles. P and R^2^ values are represented.

We examined the frequency of these aberrations in circulating lymphocytes of 50 patients, more than 12 years after radiation therapy and previously published^11, 26–29^ (Table 2), to validate our observation that the specific configuration of DCs and telomere deletions are persistent aberrations after successive cell divisions. We found a high frequency of DC with both centromeres juxtaposed (62.5%) (Fig. 5A,B). These DC were not accompanied by an AC and may have been derived from the bone marrow after more than three divisions. We observed some lymphocytes with DCs and RC accompanied by AC (+/+), reflecting their presence at the time of irradiation, as well as their longevity. Circulating lymphocytes of patients had considerably and significantly more telomere deletions than the circulating lymphocytes of healthy donors (Fig. 5C, p-value < 10^−15^, Fisher’s exact test). Strikingly, a subset of patients demonstrated a high frequency of telomere deletions associated with the presence of DC and AC. Of note, the frequency of CAs found many years after radiation therapy did not correlate with the clinical characteristics of patients nor their treatment modalities.

**Table 2:** Clinical characteristics, modalities of treatment and follow-up of treated Hodgkin lymphoma patients.

Characteristics	Patients (N = 50)
Age at treatment (median and range)	29 (18–68)
Age at CA scoring (median and range)	41 (27–79)
Male/Female ratio	23/27 (0.85)
Stage	
Early stage	42 (84%)
Advanced stage	8 (16%)
Treatment	
Chemotherapy	50 (100%)
Radiation therapy	50 (100%)
Total radiation dose (Gy)	36 (35, 4–36,8)
Follow-up(median and range)	12 (3–30)

## Discussion

The association between cancer risk and the frequency of CA in populations exposed to radiation was previously demonstrated^2, 3, 9, 26^. The scoring of CA for the estimation of the dose received, an important parameter for estimating the associated risk, is a powerful approach, in particular in the absence of or the impossibility to perform physical dosimetry^10^. The transmission of these aberrations has been previously investigated using conventional cytogenetic techniques (FPG)^13–17, 19, 30^ and FISH^14, 20, 21^. However, investigation of the transmission of ACs was performed using conventional cytogenetic techniques (uniform staining) without any distinction between the different types of ACs, and their transmission remains unclear.

Using TC staining and extended lymphocyte culture times, we demonstrate, for the first time, that the yield of DCs decreased 50.12% between M1 and M2, much closer to the theoretical rate (50%)^13, 31^ than found in all other studies^15, 17, 20, 32^. Extended lymphocyte culture times permitted to resolve of, not only the mitotic lag of exposed lymphocytes observed at 50 h, but also the difference in the proliferation indices between the three donors.

TC staining facilitates the detection of certain specific DC configurations, especially when both centromeres are very close to each other (DC-C) or when the centromere is very near the telomere sequences (DC-B). The detection of these aberrations is very difficult using conventional staining and they can be easily mistaken for translocations by M-FISH or chromosome painting, because centromeric regions are not labelled by these probes. This work demonstrates that these DC-B and DC-C represented only 30% of all DCs scored in M1 cells, but more than 70% in ≥M3, showing the higher transmission rate of these DC configurations. In agreement, the scoring of persisting DCs in circulating lymphocytes of patients treated by radiation therapy showed that 82% of detected DCs were characterized by both centromeres or the centromere and telomeres in close proximity. After radiation therapy, the restauration from lymphopenia occurs through increased activity of stem cells or the migration of lymphocytes into the blood stream from non-irradiated areas^7^. These new lymphocytes carry these specific DC configurations induced by irradiation of the stem cells. More than three divisions are necessary to produce new lymphocytes. In addition, the most clonal DC present in tumor cells or in tumor cell lines were those with both centromeres or the centromere and telomeres in close proximity (data not shown). Altogether, the *in vitro* and *in vivo* data demonstrate the higher transmission rate of DCs with both centromeres close together. Two possible mechanisms may explain the formation of these DC configurations. In the first, telomere deletion, observed after *in vitro* and *in vivo* exposure, initiates breakage-fusion-bridge events^33–36^ and induces chromosome fusion and DC formation. Hybridization with subtelomeric and M-FISH probes could confirm this hypothesis. The second possible mechanism consists of the complete loss of a chromosome arm after DSB in the pericentromeric region and the formation of a DC with the centromeres in close proximity. Close proximity of centromeres has been proposed to result in the stability of DCs, either because it promotes inactivation of one of the centromeres and converts the DC into a stable monocentric chromosome^35, 37–42^, or because it reduces the likelihood that the two centromeres will be pulled in opposite directions during anaphase^33, 43, 44^.

The consequences of both mechanisms are similar: stabilization of the DC. However, breakage/fusion/bridge events conserve a normal karyotype without loss and gain. Nevertheless, the DC resulting from two pericentromeric breaks were associated with the complete loss of a chromosome arm and gene copy number changes. This would be an important step in the mutagenesis that leads to oncogenesis. Recently, study in yeast has shown that DCs lacking telomere fusion preferentially break in pericentromere regions^45^.

Concerning the transmission of CRs, the decline of CRs between M1 and M2 was only 19.05% and the F-ratio decreased with cell division (5.41, 3.19 and 3.39). The F-ratio obtained at M1 in this study is in agreement with those described previously^25, 46, 47^. These data are in agreement with previous reports^15, 48, 49^ showing the persistence of small rings during cell divisions. *In vivo* data corroborate the efficiency of transmission of small CRs during cell division (more than 77% of detected CR) and the familial transmission of mosaic ring syndrome^50, 51^. The detection of small CRs is very difficult by conventional staining and they can be easily mistaken for acentric chromosomes. These data confirm the importance of taking into account DCs and CRs in biological dosimetry^1^ and TC staining for the reliable and robust scoring of CAs as, not only a new tool for biological dosimetry^52^, but also for assessing prenatal and congenital cases, as well as tumor cells.

Centromeric sequences are essential for the accurate segregation of chromosomes to daughter cells during mitosis, and induced ACs should be rapidly lost when the nuclear membrane breaks down at mitosis^53^. Here, we investigated, first the transmission of all ACs in M2 and ≥M3 and second, the transmission of each kind of AC. The reduction of all ACs between M1 and M2 was approximately 30%. This rate is in accordance with previous reports^15, 16^. The use of TC staining, followed by M-FISH, permits precise scoring of each type of ACs, as well as the presence of paired ACs in M2 and ≥M3 cells. We demonstrate the presence of paired AC (+/+), ACs resulting from a fusion event generally accompanying the formation of a DC or CR, in M2 and ≥M3 cells. The frequency of AC (+/+) would be expected to increase in M2 cells. However, our results show that the frequency of AC (+/+) in M2 declined by 35%, meaning that only 32.5% of AC (+/+) in M1 cells could be transmitted to M2 cells and that most AC (+/+) (67.5%) were eliminated. A possible mechanism for the decrease of AC (+/+) in M2 cells is the conversion of AC (+/+) to a micronucleus^54^ and inclusion of two or more ACs into one micronucleus at a higher radiation dose^55, 56^. Furthermore, we demonstrate that AC (+/+) that persisted and replicated in M2 cells were formed after one or two breaks in the pericentromeric region (Figs 2E and 4A–C). The same mechanism may be responsible for the transmission of small rings associated with the formation of paired AC (+/+) (Figs 2E-c and 4A–C). We demonstrate that only AC (+/+) resulted to the breakpoint near or in centromeric region were able to replicate at least once. In addition, using the CBMN assay, Minissi *et al*.^57^ provided evidence that reduced polar distance at anaphase may favor the re-incorporation of lagging chromosomes or fragments into the daughter nuclei^57^. The transmission of AC (+/+), that represent most ACs in M1 cells, has yet to be investigated. Of note, we found centromere sequences in some replicated AC (+/+) (Fig. 2Db) showing that the breakpoint was in centromere region.

Similarly, AC (−/−), interstitial AC, were able to replicate, and paired AC (−/−) were formed. Their frequency increased by 10% in M2, such that only 55% of AC (−/−) in M1 cells were transmitted to M2 cells. Taken together, the collective decline of AC (+/+) and AC (−/−) frequency was 27% in M2, resulting in only 36.5% transmission to M2 cells. These data are in agreement with a previously published study^54^, showing that 40% of ACs observed in irradiated lymphocyte cultures after 48 h, are not observed as micronuclei after 72 h. These data must be supplemented with data obtained using the CBMN assay associated with TC staining (micronucleus with or without TC sequences and the number of sequences) to better understand the mechanism of transmission of these aberrations. Of note, in ≥M3 cells, only a few of AC (+/+) and AC (−/−) pairs observed in M2 cells were able to produce a new pair of ACs. Similarly, the *in vivo* study demonstrates the absence of paired AC (+/+) and AC (−/−) associated with the presence of specific configurations of DC or CR in circulating lymphocytes of exposed patients. The observed AC (+/+) and AC (−/−) were associated with the presence of DCs or CRs in directly irradiated lymphocytes (M1) and not available for bone marrow differentiation.

Sequential analysis did not reveal the presence of paired AC (+/−) at M2 or later, showing that the observed AC (+/−) in M2 cells was formed during the last mitotic division cycle, since no replication was observed. The decline of AC (+/−) frequency between M1 and M2 was approximately 50%. However, the decline was less between M2 and ≥M3, confirming that the observed AC (+/−) in M2 and M3 cells were not transmitted. The scoring of the number of telomere sequences in micronuclei at 72 h could confirm the elimination of AC (+/−).

Telomere deletion (Chr +/−) was detected only by TC staining. This aberration is not considered for biological dosimetry studies nor the estimation of the background of CA in the general population. Our data indicate that the frequency of Chr (+/−) in circulating lymphocytes of 68 healthy donors was not age-dependent and showed high inter-individual variation. These data underline the importance of re-evaluating the frequency of spontaneous unstable CAs by TC staining and introducing this aberration into estimations of the background of the general population and the risk associated with IR exposure. After 4 Gy exposure, the rate of this aberration in M2 cells was higher than that observed in M1 cells (+34%). In ≥M3 cells, this aberration represented one of the major persistent aberrations. In the *in vivo* study, the higher frequency of Chr(+/−) observed in circulating lymphocytes of patients treated by radiation therapy confirm the important role of this aberration. The scoring of this aberration in retrospective biological dosimetry, as well as in the follow-up of populations exposed to ionizing radiation, will be of great interest, not only for more precise dose estimations, but also for the investigation of mechanisms of radiation induced chromosomal instability and the estimation of the associated risks in the monitoring of populations exposed to ionizing radiation.

Here, we demonstrate that the frequency of CAs in M1 cells increased with time after mitogen stimulation, which is confirmed by previous data^32^ demonstrating that the frequency of CAs in the same post-irradiation division were higher in cultures at later sampling times. We also demonstrated the same trend for all CA, including AC (−/−), AC (+/−) and Chr (+/−). This approach could be used in retrospective biological dosimetry in order to estimate the absorbed dose with more precision and to define the risk associated.

Donor-dependent giant cells represented approximately 10% of scored M2 cells and less for M3 cells and may be related to blocked karyokineis and the formation of tetraploid cells. The giant cells all shared a high frequency of centromeric breakpoints, as well as telomere deletions. These CAs appear to be a driving force for the generation of chromosomal instability (Fig. 4). Of note, the presence of chromosome pulverization in M2 giant cells may be related to chromothripsis^58, 59^.

## Conclusion

The improvement in the detection of CAs using TC staining permits the reevaluation of the transmission of, not only DCs and CRs with higher efficiency, but also that of ACs. We demonstrate, for the first time, the higher efficiency of transmission of DC configurations with centromere–centromere and centromere-telomere sequences. These DC configurations were associated mostly with the presence of paired acentric chromosomes in M2 cells. Similarly, the small RCs detected in M2 cells, accompanied by paired AC (+/+), represented most scored RCs in M2 and ≥M3 cells. An *in vivo* study confirmed the higher transmission of these DC or CR configurations. These DC and RC configurations are not detected by conventional staining nor by molecular cytogenetic approaches. TC staining may not only be a new tool for biological dosimetry immediately after radiation exposure, but also for retrospective biological dosimetry and the monitoring of populations exposed to genotoxic agents.

In light of these data, it will be necessary to first reevaluate the background of CAs in general population using the nomenclature proposed herein and then to validate these approaches in a large cohort of populations exposed to genotoxic agents, including ionizing radiation (high and low doses) and performed retrospective biological dosimetry using TC staining to provide precise cytogenetic biological dosimetry and refine the associated estimated risk. It will be important to determine the breakpoints of “stable and persisted DCs” with higher precision and the chromosomes implicated in these aberrations.

## Materials and Methods

### Ethic Statement

This study was performed in accordance with the ethical guidelines of the Helsinki declaration of 1975 (revised in 2013). The samples, the medical records and all methods used in our study have been approved by the Ethics Committee of Gustave Roussy Cancer Campus University Paris Sud (approval number 97-06). All healthy donors and patients signed an informed consent form consistent with institutional review board guidelines.

### Materials and irradiation procedure

Peripheral blood samples were obtained from three healthy donors (one female and two males, mean age 43 years (36–52 years)) and exposed to gamma irradiation using an IBL637 ^137^Cs irradiator at 4 Gy at a dose rate of 0.61 Gy/min.

A cohort of 50 Hodgkin lymphoma patients treated in the department of hematology and radiation therapy at Gustave Roussy Cancer Campus were included in this study. All patients received four to six cycle of chemotherapy. Involved- or extended field of radiation therapy was delivered (35.4–36.8 Gy). Blood samples were obtained during the follow-up more than 12 years after treatment. These samples were used to validate the *in vitro* data. Clinical characteristics, as well as treatment modalities, are shown in Table 2.

A cohort of 68 healthy donors (mean age 29.36 years (2–56.6years) was used as a control. None of the donors had been recently exposed to ionizing radiation for diagnostic or therapeutic purposes.

## Methods

### Metaphase preparation

Blood lymphocytes were cultured in RPMI 1640 medium (Gibco, Grand Islands, NY) complemented with 10% fetal bovine serum (Eurobio, Courtaboeuf, France) in the presence of 1.5% phytohaemagglutinin (PHA) (Gibco, Grand Island) and 1% bromodeoxyuridine (Sigma-Aldrich) at 5.0 mg/ml, for 50, 72, and 96 h in a humidified 5% CO2 incubator at 37 °C for *in vitro* irradiation of blood samples. The same culture conditions were used for blood samples of treated patients and healthy donors, but only one culture time was performed (48–50 h).

Metaphase preparations were performed using standard procedures^60^. Slides were prepared and stored at −20 °C until use.

### Telomere and centromere staining

Telomeres and centromeres were stained using the Q-FISH technique with a Cy-3-labelled PNA probe specific for TTAGGG for telomeres and a FITC-labeled PNA probe specific for centromere sequences (both from Panagene, Daejon, South Korea) as described in M’kacher *et al*.^23^. Image acquisition and analysis were performed using Isis software, (version 3.9.1, MetaSystems, version 3.9.1).

## M-FISH

Staining was performed using multicolor FISH probes (MFISH 24XCyte, Metasystems) according to the protocol provided by the manufacturers on the same slides used for TC staining. Metaphases images were captured using image analyzer Metasystems/AutoCapt software.

### Chromosomal aberration scoring

Two slides were used for each cell cycle of culture at 0 and 4 Gy. For each condition, an average of 100 metaphases or more were counted for each slide. The first slide was stained with FPD prior to TC staining and the second was subjected only to TC staining. The use of these two approaches permits determination of the cell division. FPD is a conventional technique that allows the determination of the cell division status of each scored cell^1^ due to the incorporation of BrdU by 2-strand substitution. PNA probe staining distinguishes the 1^st^ metaphase from the 2^nd^ and 3^rd^ metaphases due to cross hybridization of the FITC signal^23^.

CAs were detected based on the detection of centromeric regions and telomeric sequences. We detected dicentric (DC) and centric ring (CR) chromosomes and acentric chromosomes (ACs) with four telomeres (AC (+/+)), resulting from a fusion event generally accompanying the formation of a DC or a CR. We also detected ACs with two telomeres (AC (+/−)), representing terminal deletions, as well as acentric fragments without telomeres (AC (−/−)), representing interstitial deletions. Chromosomes with telomere deletions in one or both arms were also scored (chr +/− or chr −/−) (Fig. 1A).

Specific DC and CR were analyzed: DC-A with the two centromeres far apart; DC-B with one centromere close to a telomere, and DC-C with two centromeres in close proximity (Fig. 1a). For the DC-C configuration, the distance between two centromeres was measured using Isis software (MetaSystems, version 3.9.1) and the maximum distance defining this configuration was 1.5 μm

The DC and CR accompanied by an AC (+/+) or two AC (+/−) were classified as complete or incomplete when the ACs were missing.

The scoring of CA was performed only on complete metaphases with 46 centromeres.

### Slide scanning and Metaphase acquisition

Images of metaphase cells were acquired using automated acquisition module Autocapt software (MetaSystems, version 3.9.1) and a ZEISS Plan-Apochromat 63x/1.40 oil and CoolCube 1 Digital High Resolution CCD Camera. Settings for exposure and gain remained constant between captures. The analysis was performed using TCscore as described in M’kacher *et al*.^23^ and Isis software (MetaSystems, version 3.9.1).

### Statistical analysis

Extending the lymphocyte culture time from 50 to 72 and 96 h, permits the analysis of the frequency of CA in successive post-irradiation cell divisions, as well as the transmission of these aberrations, taking into account the delay in cell cycle progression of the aberrant cells. The rate of transmission of various CA was performed for different cell divisions at various culture times.

The proportion of cells in each cell division was determined using randomly chosen metaphases and a proliferation index (PI) was calculated where PI = (1 x the number of first division metaphase) + (2 x the number of second division metaphases) + (3 x the number of third or more division metaphases)]/number of cells scored. All plots were created using R and the gplots 2.1.0 package. The 95% confidence interval limits were calculated using the exact Poisson method, using the chi 2 distribution function.

Linear regression was performed to fit the data (slope and intercept) using the least squares method. The significance test for linear regression (p-value and squared-R) were performed using the lm() R command including the Fisher-Test for regression. The ANOVA tests were used to compare CA in irradiated cells versus controls and at different divisions and circulating lymphocytes of treated patients versus healthy donors

Variations in the frequency of the aberration scored were considered to be significant when the confidence intervals did not overlap or when the uniformly most powerful (UMP) unbiased test (see Lehmann and Romano, 2005, p.125) gave a p-value with the null hypothesis with the ratio of the compared count with time at risk equal to 1 (no significant difference). The ratio rate test was performed using Test library in R software.

## Electronic supplementary material

Supplementary Information
