# Supplementary material for: Transmission of Induced Chromosomal Aberrations through Successive Mitotic Divisions in Human Lymphocytes after In Vitro and ﻿In﻿ Vivo Radiation
Source: Sci Rep. 2017 Jun 12;7:3291. doi: 10.1038/s41598-017-03198-7 (PMC5468351; doi:10.1038/s41598-017-03198-7)

**Transmission of Induced Chromosomal Aberrations through Successive Mitotic Divisions in Human Lymphocytes after In Vitro and Vivo Radiation**

Akram Kaddour^1-2^, Bruno Colicchio^3^, Diane Buron^1^, Elie El Maalouf^3^, Eric Laplagne^4^, Claire Borie^5^, Michelle Ricoul^1^, Aude Lenain^1^, William M. Hempel^1^, Morat Luc^1^, Mustafa AL Jawhari^1^, Corina Cuceu^1^, Leonhard Heidingsfelder^6^, Eric Jeandidier^7^, Georges Deschenes^8^, Alain Dieterlen^3^, Michelle EL May^2^, Theodore Girinsky^9^, Annelise Bennaceur-Griscelli^5^, Patrice Carde^10^, Laure Sabatier^1^, Radhia M’kacher^1-11*^

^1^ Laboratory of Radiobiology and Oncology, IRCM, DSV, CEA, Fontenay aux Roses, France

^2^ Tunis El Manar University, School of Medicine, Tunis, Tunisia

^3^ Laboratoire MIPS Groupe IMTI Université de Haute-Alsace, Mulhouse, France

^4^ Pole Concept, Paris, France

^5^APHP-Hopital Paul Brousse Université Paris Sud/ESteam Paris Inserm UMR 935, Villejuif, France

^6^ MetaSystems GmbH, Robert-Bosch-Str. 6 D-68804. Altlussheim, Germany

^7^Service de Génétique Groupe Hospitalier de la Région de Mulhouse et Sud Alsace 68070 Mulhouse, France

^8^ Nephrology Department, APHP-Hopital Robert Debré, Paris, France

^9^Department of Radiation Oncology, Gustave Roussy Cancer Campus, Villejuif, France

^10^Department of Hematology, Gustave Roussy cancer Campus, Villejuif, France

^11^Cell Environment, Paris, France

*Corresponding author: Cell Environment Radiobiology and Oncology Section, APHP-Hopital Paul Brousse Université Paris Sud Villejuif France

Email: [radhia.mkacher@gmail.com](mailto:radhia.mkacher@gmail.com)

**Legends for Supplementary Tables and Figures**

**Table S1-** Frequencies of CAs in circulating lymphocytes of each donors following telomere and centromere staining according cell division and time of culture (A) donor 1 (B) donor 2 and (C) donor 3

**Table S2-** Human lymphocyte proliferation through cell division in 50h, 72h and 96h culture after exposure to 4 Gy and compared to nonirradiated lymphocytes

**Table S3:** Frequencies and distribution of DCs in M1, M2 and M3 cells after 4 Gy irradiation using telomere and centromere staining

**Figure S1:** The frequency of CAs of M2 and M3 cells after 50, 72 and 96 h in culture after 4Gy irradiation. (A) The frequency of DCs and CR in M2 cells (B) The frequency of AC (+/-), AC (+/+), AC (-/-) and Chr (+/-) in M2 cells. (C) The frequency of DC and CR in M3 cells (E) The frequency of AC (+/-), AC (+/+), AC (-/-) and Chr (+/-) in M3 cells.

Supplementary Table S1

| 1. **Donor 1** | | | | | | | | | | | | | | | | |
| --- | --- | --- | --- | --- | --- | --- | --- | --- | --- | --- | --- | --- | --- | --- | --- | --- |
| **Cell Division** | **M1** | | | | | | **M2** | | | | | | **M3** | | | |
| **Time of culture (h)** | **50** | | **72** | | **96** | | **50** | | **72** | | **96** | | **72** | | **96** | |
| Scored cells | 196 | | 18 | | 18 | | 53 | | 51 | | 33 | | 44 | | 207 | |
| Total DC (/cell) | 293 | (1,49) | 35 | (1,94) | 21 | (1,17) | 41 | (0,77) | 40 | (0,78) | 16 | (0,48) | 12 | (0,27) | 40 | (0,19) |
| Total RC (/cell) | 57 | (0,29) | 9 | (0,50) | 8 | (0,44) | 13 | (0,25) | 8 | (0,16) | 10 | (0,30) | 0 | (0,00) | 15 | (0,07) |
| **Total DC + RC (/cell)** | 350 | (1,79) | 44 | (2,44) | 29 | (1,61) | 54 | (1,02) | 48 | (0,94) | 26 | (0,79) | 12 | (0,27) | 55 | (0,27) |
| **Total AC (/cell)** | 503 | (2,57) | 54 | (3,00) | 35 | (1,94) | 72 | (1,36) | 130 | (2,55) | 43 | (1,30) | 7 | (0,16) | 40 | (0,19) |
| AC(+/+) (%AC) | 345 | (69%) | 35 | (65%) | 23 | (66%) | 44 | (61%) | 59 | (45%) | 18 | (42%) | 2 | (29%) | 27 | (68%) |
| AC (-/−)(%AC) | 109 | (22%) | 11 | (20%) | 8 | (23%) | 23 | (32%) | 58 | (45%) | 19 | (44%) | 2 | (29%) | 4 | (10%) |
| AC(+/-)(%AC) | 49 | (10%) | 8 | (15%) | 4 | (11%) | 5 | (07%) | 13 | (10%) | 6 | (14%) | 3 | (43%) | 9 | (23%) |
| **Chr(+/-) (/cell)** | 40 | (0,20) | 14 | (0,78) | 13 | (0,72) | 4 | (0,08) | 26 | (0,51) | 18 | (0,55) | 10 | (0,23) | 32 | (0,15) |
| Complete DC+CR (/cell) | 325 | (1,66) | 35 | (1,94) | 19 | (1,06) | 16 | (0,30) | 19 | (0,37) | 7 | (0,21) | 1 | (0,02) | 7 | (0,03) |
| Incomplete DC+CR (/cell) | 25 | (0,13) | 9 | (0,50) | 10 | (0,56) | 38 | (0,72) | 29 | (0,57) | 19 | (0,58) | 11 | (0,25) | 48 | (0,23) |

1. **Donor 2**

| **Cell Division** | **M1** | | | | | | **M2** | | | | | | **M3** | | | |
| --- | --- | --- | --- | --- | --- | --- | --- | --- | --- | --- | --- | --- | --- | --- | --- | --- |
| **Time of culture (h)** | **50** | | **72** | | **96** | | **50** | | **72** | | **96** | | **72** | | **96** | |
| Scored cells | 107 | | 17 | | 3 | | 67 | | 94 | | 43 | | 80 | | 86 | |
| Total DC (/cell) | 163 | (1,52) | 33 | (1,94) | 4 | (1,33) | 49 | (0,73) | 96 | (1,02) | 22 | (0,51) | 30 | (0,38) | 19 | (0,22) |
| Total RC (/cell) | 33 | (0,31) | 5 | (0,29) | 4 | (1,33) | 15 | (0,22) | 28 | (0,30) | 6 | (0,14) | 8 | (0,10) | 7 | (0,08) |
| **Total DC + RC (/cell)** | 196 | (1,83) | 38 | (2,24) | 8 | (2,67) | 64 | (0,96) | 124 | (1,32) | 28 | (0,65) | 38 | (0,48) | 26 | (0,30) |
| **Total AC (/cell)** | 309 | (2,89) | 46 | (2,71) | 5 | (1,67) | 130 | (1,94) | 182 | (1,94) | 59 | (1,37) | 30 | (0,38) | 32 | (0,37) |
| AC(+/+) (%AC) | 206 | (67%) | 26 | (57%) | 3 | (60%) | 79 | (61%) | 127 | (70%) | 34 | (58%) | 16 | (53%) | 27 | (84%) |
| AC (-/−) (%AC) | 77 | (25%) | 6 | (13%) | 0 | (00%) | 39 | (30%) | 49 | (27%) | 23 | (39%) | 13 | (43%) | 3 | (09%) |
| AC(+/-) (%AC) | 26 | (08%) | 14 | (30%) | 2 | (40%) | 12 | (09%) | 6 | (03%) | 2 | (03%) | 1 | (03%) | 2 | (06%) |
| **Chr(+/-) (/cell)** | 20 | (0,19) | 8 | (0,47) | 3 | (1,00) | 28 | (0,42) | 57 | (0,61) | 14 | (0,33) | 16 | (0,20) | 25 | (0,29) |
| Complete DC+CR (/cell) | 184 | (1,72) | 27 | (1,59) | 4 | (1,33) | 34 | (0,51) | 44 | (0,47) | 11 | (0,26) | 3 | (0,04) | 4 | (0,05) |
| Incomplete DC+CR (/cell) | 12 | (0,11) | 11 | (0,65) | 4 | (1,33) | 30 | (0,45) | 80 | (0,85) | 17 | (0,40) | 35 | (0,44) | 22 | (0,26) |

1. **Donor 3**

| **Cell Division** | **M1** | | | | | | **M2** | | | | | | **M3** | | | |
| --- | --- | --- | --- | --- | --- | --- | --- | --- | --- | --- | --- | --- | --- | --- | --- | --- |
| **Time of culture (h)** | **50** | | **72** | | **96** | | **50** | | **72** | | **96** | | **72** | | **96** | |
| Scored cells | 151 | | 40 | | 10 | | 54 | | 86 | | 25 | | 53 | | 100 | |
| Total DC (/cell) | 249 | (1,65) | 101 | (2,53) | 13 | (1,30) | 66 | (1,22) | 62 | (0,72) | 19 | (0,76) | 32 | (0,60) | 26 | (0,26) |
| Total RC (/cell) | 44 | (0,29) | 14 | (0,35) | 1 | (0,10) | 20 | (0,37) | 21 | (0,24) | 7 | (0,28) | 14 | (0,26) | 12 | (0,12) |
| **Total DC + RC (/cell)** | 293 | (1,94) | 115 | (2,88) | 14 | (1,40) | 86 | (1,59) | 83 | (0,97) | 26 | (1,04) | 46 | (0,87) | 38 | (0,38) |
| **Total AC (/cell)** | 401 | (2,66) | 150 | (3,75) | 26 | (2,60) | 146 | (2,70) | 160 | (1,86) | 46 | (1,84) | 19 | (0,36) | 18 | (0,18) |
| AC(+/+) (%AC) | 289 | (72%) | 99 | (66%) | 16 | (62%) | 94 | (64%) | 93 | (58%) | 30 | (65%) | 5 | (26%) | 3 | (17%) |
| AC (-/−) (%AC) | 75 | (19%) | 25 | (17%) | 10 | (38%) | 41 | (28%) | 59 | (37%) | 9 | (20%) | 13 | (68%) | 14 | (78%) |
| AC(+/-) (%AC) | 37 | (09%) | 26 | (17%) | 0 | (00%) | 11 | (08%) | 8 | (05%) | 7 | (15%) | 1 | (05%) | 1 | (06%) |
| **Chr(+/-) (/cell)** | 35 | (0,23) | 16 | (0,40) | 2 | (0,20) | 14 | (0,26) | 19 | (0,22) | 3 | (0,12) | 6 | (0,11) | 17 | (0,17) |
| Complete DC+RC (/cell) | 271 | (1,79) | 100 | (2,50) | 14 | (1,40) | 41 | (0,76) | 41 | (0,48) | 12 | (0,48) | 2 | (0,04) | 0 | (0,00) |
| Incomplete DC+RC (/cell) | 22 | (0,15) | 15 | (0,38) | 0 | (0,00) | 45 | (0,83) | 42 | (0,49) | 14 | (0,56) | 44 | (0,83) | 38 | (0,38) |

|  | **Supplementary Table S2**: | | | | | | | | | | | | |
| --- | --- | --- | --- | --- | --- | --- | --- | --- | --- | --- | --- | --- | --- |
|  | **Time of culture** | **50h** | | | | **72h** | | | | **96h** | | | |
|  | Cell cycle phase | M1 | M2 | M3 | PI | M1 | M2 | M3 | PI | M1 | M2 | M3 | PI |
| **Donor1** | control | 56 | 37 | 6 | 1.49 | 2 | 93 | 83 | 2.45 | 3 | 92 | 326 | 2.77 |
|  | irradiated at 4Gy | 222 | 49 | 0 | 1.18 | 13 | 50 | 16 | 2.04 | 0 | 18 | 124 | 2.88 |
| **Donor2** | control | 82 | 104 | 3 | 1.58 | 0 | 135 | 69 | 2.34 | 0 | 34 | 242 | 2.88 |
|  | irradiated at 4Gy | 76 | 45 | 0 | 1.374 | 4 | 57 | 28 | 2.27 | 3 | 44 | 103 | 2.67 |
| **Donor3** | control | 54 | 49 | 0 | 1.47 | 4 | 214 | 138 | 2.38 |  |  |  |  |
|  | irradiated at 4Gy | 196 | 57 | 0 | 1.22 | 40 | 134 | 54 | 2.06 | 7 | 23 | 107 | 2.73 |
|  | PI: proliferation index | | |  |  |  | | | | | | | |

**Supplementary Table S3**:

| **Cell division** | **Cell scored** | | **No DC** | | **DC/cell** | **DC distribution** | | | | | | | | | | σ2/y | **U** | **Poisson Distribution** |
| --- | --- | --- | --- | --- | --- | --- | --- | --- | --- | --- | --- | --- | --- | --- | --- | --- | --- | --- |
|  |  |  | |  | | **0** | **1** | **2** | **3** | **4** | **5** | **6** | **7** | **8** | **9** |  |  |  |
| M1 | 466 | 755 | | 1,62 | | 76 | 166 | 115 | 60 | 29 | 11 | 0 | 0 | 1 | 0 | 0.94 | -0.97 | Yes |
| M2 | 327 | 254 | | 0,77 | | 171 | 86 | 50 | 12 | 8 | 0 | 0 | 0 | 0 | 0 | 1.28 | 3.61 | No |
| M3 | 347 | 102 | | 0,29 | | 266 | 63 | 14 | 1 | 2 | 0 | 0 | 0 | 0 | 0 | 1.28 | 3.61 | No |

**Supplementary Figure S1**:


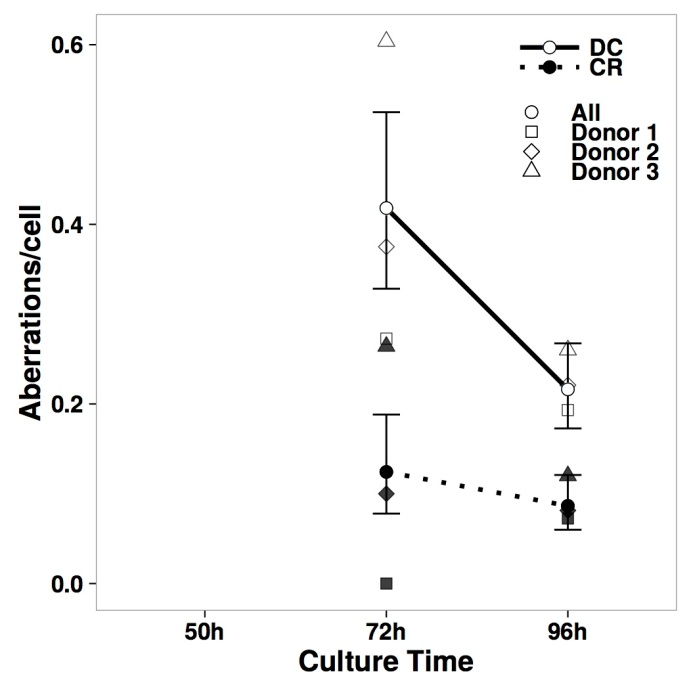

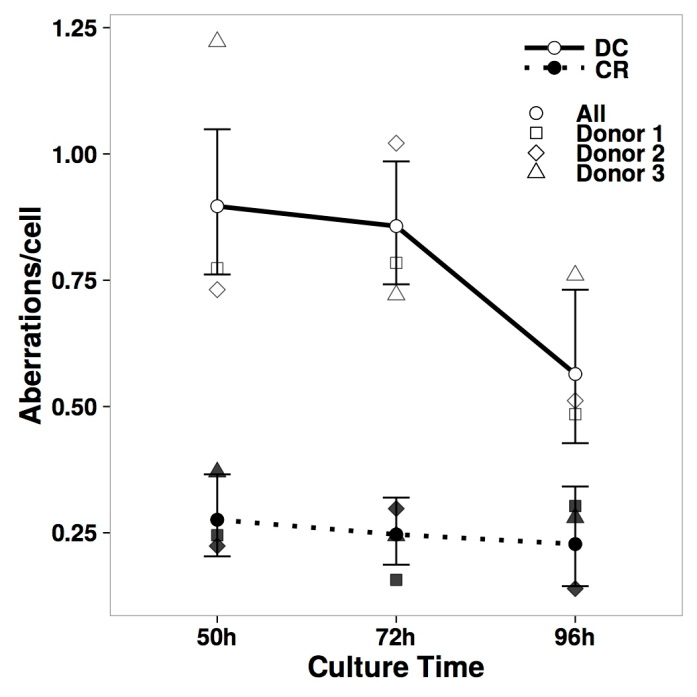


A

B

D

C


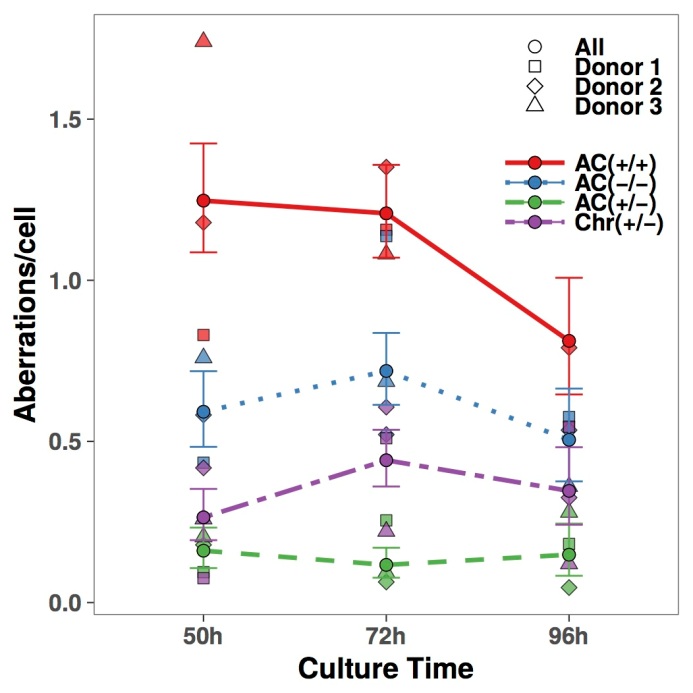

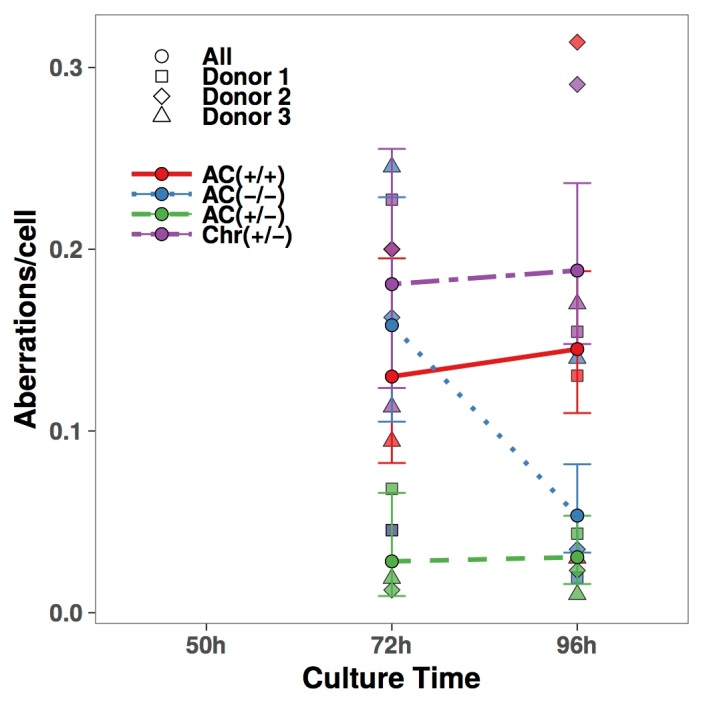

Supplement: Supplementary file 1 — Supplementary Information [file 41598_2017_3198_MOESM1_ESM.doc]
